# A Rapid Visual Detection Method for *Fasciola hepatica* Based on RAA-CRISPR/Cas12b

**DOI:** 10.3390/ani16071093

**Published:** 2026-04-02

**Authors:** Jiangying Li, Tao Zhang, Jingkai Ai, Zijuan Zhao, Zhi Li, Yong Fu, Dan Jia, Hong Duo, Xiuying Shen, Ru Meng, Yingna Jian, Xueyong Zhang

**Affiliations:** 1 The Academy of Animal and Veterinary Sciences, Qinghai University, Xining 810016, China; ljiangying@126.com (J.L.); 18656899971@163.com (T.Z.); jingkaiaitq@outlook.com (J.A.); 15540892826@163.com (Z.Z.); lizhi19880717@163.com (Z.L.); qhfuyong@163.com (Y.F.); jiadan1995@163.com (D.J.); 13639716578@163.com (H.D.); qhshenxiuying@163.com (X.S.); 2 Qinghai Provincial Key Laboratory of Pathogen Diagnosis for Animal Disease and Green Technical Research for Prevention and Control, Xining 810016, China; 3 Xining Animal Disease Prevention and Control Center, Xining 810016, China; mr-0522@163.com

**Keywords:** *Fasciola hepatica*, RAA, CRISPR/Cas12b, visual detection, test strip

## Abstract

Fascioliasis, a globally prevalent zoonosis, severely threatens public health and livestock security. Current diagnostic approaches, hindered by the need for sophisticated instrumentation and specialized expertise, are inadequate for on-site surveillance in resource-constrained settings. This study developed a rapid, visual detection assay for *Fasciola hepatica* via recombinase-aided amplification (RAA) integrated with CRISPR/Cas12b, addressing critical equipment and operational constraints. Targeting a specific mitochondrial DNA fragment of *F. hepatica*, recombinant plasmid standards were constructed, RAA primers and sgRNA optimized, and three detection modalities (real-time fluorescence, UV lamp, test strip) integrated. Clinical validation against PCR demonstrated 45 min turnaround time, *F. hepatica*-specific positivity, and real-time fluorescence sensitivity of 2.6 copies/μL. Results showed high concordance with PCR and qPCR, with substantially reduced assay duration and streamlined workflow. This highly sensitive, specific, multi-visualized method overcomes limitations of conventional techniques, offering an efficient, field-deployable tool for fascioliasis surveillance and control in grassroots and pastoral regions.

## Simple Summary

*Fasciola hepatica* parasitizes the hepatic bile ducts of animals such as cattle and sheep, and can also infect humans, posing a dual threat to the livestock industry and public health. However, existing detection techniques have drawbacks such as insufficient sensitivity or reliance on sophisticated instruments, making it difficult to meet the practical need for rapid and accurate detection. This study aimed to establish a convenient and reliable detection method for *F. hepatica*. By integrating the recombinase-aided amplification (RAA) with the CRISPR/Cas12b system, the detection system was optimized, and three visual detection modes, namely real-time fluorescence, ultraviolet lamp, and test strip, were constructed. Moreover, the specificity, sensitivity, repeatability, and clinical reliability of the established and optimized method were evaluated, providing an effective tool for the early diagnosis of *F. hepatica*.

## 1. Introduction

*Fasciola hepatica* is a zoonotic parasite with a worldwide distribution [1], posing a serious threat to the livestock industry and public health [2]. This parasite mainly inhabits the hepatic bile ducts of various herbivores such as cattle and sheep, as well as humans, causing fascioliasis [3,4]. The disease can lead to digestive disorders and liver function damage in the host, and in severe cases, it can even cause death [5,6]. Currently, diagnostic approaches for *F*. *hepatica* mainly rely on etiological detection (microscopic examination of fecal eggs and post mortem examination), immunological diagnosis (ELISA and IHA), and molecular biological methods (PCR and qPCR) [7,8,9]. Among these, microscopic examination of fecal eggs is simple to perform but has low sensitivity, and post mortem examination is only applicable for diagnosing after death, making it unsuitable for in vivo and large-scale screening. Although immunological methods can enable early detection, they suffer from issues such as cross-reactions (due to high antigenic homology with closely related parasites like *Fasciola gigantica*) and a delay in antibody detection [10]. Although PCR and qPCR technologies can accurately detect positive samples, they have high requirements for experimental equipment and operators. Moreover, the experimental procedures are complex and time-consuming. Therefore, a rapid, sensitive, and specific diagnostic method is urgently needed for the surveillance and control of fascioliasis.

To address these limitations, we combined recombinase-aided amplification (RAA) and CRISPR-mediated specific recognition technologies to provide an innovative direction for the rapid detection of *F. hepatica*. RAA is a novel isothermal nucleic acid amplification technology that has emerged in recent years [11]. Through the coordinated action of recombinase, single-strand-binding protein, and DNA polymerase, it can complete nucleic acid amplification within 30 min under isothermal conditions of 37–42 °C, eliminating the need for complex thermal-cycling equipment [12,13]. The CRISPR/Cas12b system, as a new-generation gene-editing and detection tool, has a core protein, AapCas12b (derived from *Acidothermus cellulolyticus*) [14], which, under the guidance of sgRNA, can specifically recognize target double-stranded DNA (dsDNA) containing a PAM sequence (such as TTN) [15]. Once bound, it activates the “trans-cleavage” activity towards non-specific single-stranded DNA (ssDNA) in the system [16], enabling precise recognition of the target sequence [17].

In this study, we established an RAA-CRISPR/Cas12b detection system targeting the species-specific mitochondrial DNA of *F. hepatica*. Using three readout modes (real-time fluorescence, UV lamp visualization, and test strip), we systematically evaluated its sensitivity, specificity, and clinical performance. This study provides a practical tool for the rapid on-site diagnosis of fascioliasis and offers a reference for developing detection assays for other veterinary parasites.

## 2. Materials and Methods

### 2.1. Experimental Materials

#### 2.1.1. Sample Sources

The *F*. *hepatica* specimens were collected from the livers of sheep at an abattoir in Qinghai Province. *Fasciola gigantica*, *Fascioloides jacksoni*, *Paramphistomum cervi*, *Dicrocoelium dendriticum*, *Eurytrema pancreaticum*, *Taenia multiceps*, *Taenia crassiceps*, *Echinococcus granulosus*, *Haemonchus contortus*, *Cryptosporidium parvum* and *Giardia duodenalis* were all sourced from the laboratory of the Qinghai Academy of Animal Husbandry and Veterinary Sciences. For the clinical trial, 192 fresh ovine fecal samples were collected from farms in Xining, Qinghai Province.

#### 2.1.2. Main Reagents and Instruments

Reagents: TIANamp Genomic DNA kit (Tiangen, Beijing, China, DP304) and TIANamp Stool DNA Kit (Tiangen, Beijing, China, DP328), 2×EasyTaq^®^ PCR SuperMix (TransGen Biotech, Beijing, China, AS111), T-Vector pMD™19 (Simple) (Takara, Beijing, China, 3271), *E. coli* DH5α Competent Cell (Takara, Beijing, China, 9057), SynSor DNA/RNA Isothermal rapid amplification reagent (SynsorBio, Beijing, China, XS-R-101), DNA extraction reagent (Psaitong, Beijing, China, PS1013), SynSor AaCas 12b (C2c1) (SynsorBio, Beijing, China, XS-R-002), SynSor CRISPR ssDNA Reporter (12b-FAM) (SynsorBio, Beijing, China, XS-R-201), SynSor CRISPR ssDNA Reporter (FAM-Biotin) (SynsorBio, Beijing, China, XS-R-204), Synsor CRISPR Single-Target Detection Test Strip (FAM-Biotin) (SynsorBio, Beijing, China, XS-R-102). Instruments: qPCR instrument (Eppendorf, Hamburg, Germany); LightCycler^®^ 480 Instrument II (Roche Diagnostics GmbH, Basel, Switzerland); Dry Bath Incubator (DH200, Hangzhou Ruicheng Instrument Co., Ltd., Hangzhou, China); Gel imaging system-UV lamp (G:Box F3, SynGene, Cambridge, UK).

### 2.2. Experimental Methods

#### 2.2.1. Genomic DNA Extraction and Construction of Target Gene Standard Plasmids

Genomic DNA was extracted from the tissue of *F*. *hepatica* using the tissue DNA extraction kit (Tiangen, Beijing, China, DP304). Specific primers Fh603F/R (Table 1) were designed, and a 603 bp mitochondrial target gene fragment was amplified by PCR. The reaction procedure was as follows: pre-denaturation at 94 °C for 5 min; 35 cycles of (94 °C for 35 s, 53.5 °C for 35 s, 72 °C for 45 s); final extension at 72 °C for 10 min. After the PCR products were verified by 1.5% agarose gel electrophoresis and confirmed by sequencing, they were ligated into the pMD19-T vector (Takara, Beijing, China, 3271) and transformed into *E. coli* DH5α competent cells (Takara, Beijing, China, 9057). The positive clone plasmids were extracted, quantified, and used as quantitative standards, which were stored at −20 °C.

#### 2.2.2. sgRNA Design and Screening

Based on the selected target gene sequence of *F. hepatica*, conserved regions containing the TTN-type PAM were screened. Following the principles of GC content [18,19], secondary structure, and terminal base preference, three sgRNAs were designed (Table 1). Meanwhile, a pair of primers (FH-PCR-F/R) (Table 1) encompassing the three sgRNAs was designed for the target sequence. The product obtained after plasmid amplification was used as a template and added to the CRISPR system. After the sgRNAs were synthesized by in vitro transcription, 1 μL of sgRNA, 2 μL of AaCas12b (XS-R-002), 5 μL of 10× AaCas12b Buffer, 0.5 μL of the CRISPR ssDNA Reporter (12b-FAM) (XS-R-201), 1 μL of the template, and ddH_2_O were added to a final volume of 50 μL. The reaction was carried out at 43 °C for 15 min. The FAM fluorescence signal was monitored in real-time using a qPCR instrument. Using the fluorescence signal intensity and the fluorescence growth rate as indicators, the sgRNA with the highest activity was screened for subsequent experiments.

#### 2.2.3. Screening of RAA Primers and Optimization of Reaction Conditions

Three upstream primers (RAA-F1/F2/F3) and three downstream primers (RAA-R1/R2/R3, Table 1) were designed upstream and downstream of the sgRNA target region. Using the *F*. *hepatica* plasmid as a template, RAA cross-primer verification was carried out (9 primer combinations). The RAA reaction system consisted of 25 μL of amplification buffer A, 17 μL of ddH_2_O, 2 μL each of upstream and downstream primers, and 2 μL of plasmid. The required reagents were added to the reaction tube containing the freeze-dried bead. Finally, 2 μL of magnesium acetate was added to the cap of the reaction tube. The tube was capped, gently shaken up and down 8–10 times to mix thoroughly, and centrifuged at low speed for 10 s. The test unit tube was placed in a 42 °C Dry Bath Incubator (DH200, Hangzhou Ruicheng Instrument Co., Ltd., Hangzhou, China) for a 30 min constant-temperature reaction. After the reaction, an equal volume of DNA extraction reagent (Chloroform-isoamyl alcohol mixture) (Psaitong, Beijing, China, PS1013) was added and mixed well by pipetting. The mixture was centrifuged at 12,000 rpm for 5 min. Then, 1 μL of the upper aqueous phase of the RAA product was added to the CRISPR/Cas12b system to screen for the optimal primer pair. The effect of different reaction times on the amplification efficiency was further evaluated to determine the optimal reaction time.

#### 2.2.4. Establishment of the RAA-CRISPR/Cas12b Detection System

Real-Time Fluorescence Detection Method: Take 1 μL of the RAA product and add it to the 50 μL CRISPR/Cas12b reaction system. React at 43 °C for 15 min, and record the fluorescence curve with a qPCR instrument. The positive threshold is set as the mean fluorescence intensity of the negative control (ddH_2_O replacing the template) plus three times the standard deviation. If the fluorescence intensity of the sample exceeds the threshold and shows an upward trend, it is judged as positive.

UV Lamp Detection Method: The reaction system was the same as that of the real-time fluorescence detection method. After the reaction was completed, the reaction tube was placed under a UV lamp. If obvious fluorescence was observed, the result was judged as positive; if there was no fluorescence, it was judged as negative.

Test Strip Visual Detection Method: After the RAA reaction, 1 μL of the product was added to a CRISPR system containing 1 μL of sgRNA, 2 μL of AaCas12b, 5 μL of 10× AaCas12b Buffer, 0.2 μL of SynSor CRISPR ssDNA Reporter (FAM-Biotin) (SynsorBio, Beijing, China, XS-R-204), 1 μL of template, and ddH_2_O to make up to 50 μL. The reaction was carried out at 43 °C for 45 min. Then, 50 μL of the product was dropped into the sample application well of the Synsor CRISPR Single-Target Detection Test Strip (FAM-Biotin) (SynsorBio, Beijing, China, XS-R-102). After standing at room temperature for 5 min, the results were interpreted according to the display of the “control line (C-line) and test line (T-line)” on the test strip: both the C-line and T-line showing color development indicated a positive result, while only the C-line showing color development indicated a negative result.

#### 2.2.5. Methodological Validation

Specificity detection: Using the genomic DNA of *F*. *gigantica*, *F*. *jacksoni*, *P. cervi*, *D. dendriticum*, *E. pancreaticum*, *T. multiceps*, *T. crassiceps*, *E. granulosus*, *H. contortus*, *C. parvum* and *G. duodenalis* as templates, the three detection methods were respectively employed to evaluate the cross-reactivity.

Sensitivity detection: The plasmid standard was serially diluted to 10^5^, 10^4^, 10^3^, 10^2^, 10^1^, 10^0^, 10^−1^ copies/μL. Three technical replicates were set for each dilution gradient. The three methods were used for detection to determine the limit of detection.

Clinical sample detection: 192 ovine fecal samples were collected from sheep farms, in which sheep at slaughterhouses where liver tissues positive for *F. hepatica* had been previously identified. Briefly, approximately 10 g of fecal sample was weighed, thoroughly homogenized and macerated. After sedimentation and filtration, 200 mg of the precipitate was used for the extraction of genomic DNA. Genomic DNA was extracted from using the TIANamp Stool DNA Kit (Tiangen, Beijing, China, DP328) following the manufacturer’s instructions. The three methods developed in this study, along with qPCR [20] and PCR [21], were used for parallel testing. The positive detection rate of each method was calculated and compared.

## 3. Results

### 3.1. Construction of the Target Gene Plasmid of F. hepatica

**Figure 1:**
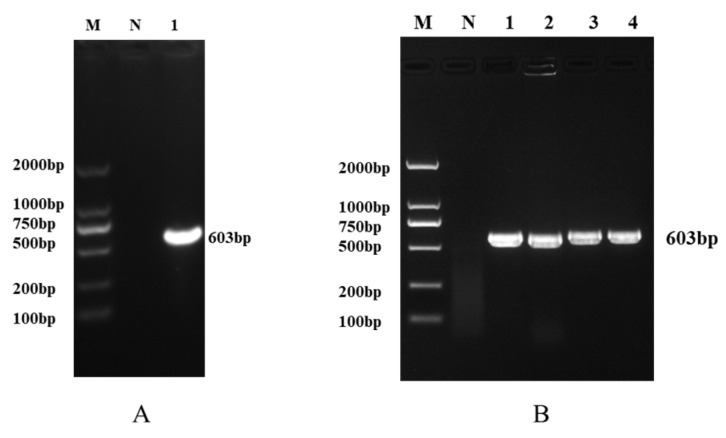
Construction of Plasmids for Target Genes of *F. hepatica*. (**A**): Electrophoresis pattern of PCR amplification of the target gene (M: DL2000 Marker; 1: Target fragment; N: Negative control). (**B**): Electrophoresis pattern of bacterial liquid PCR verification (M: DL2000 Marker; 1–4: Single colonies; N: Negative control).

The target fragment of 603 bp was obtained by PCR amplification (Figure 1A). Bacterial liquid PCR verification showed that all positive clones could amplify the corresponding fragment (Figure 1B). The sequencing results had 100% homology with the MT862417.1 sequence, indicating that the positive plasmid standard of *F*. *hepatica* was successfully constructed, with a plasmid concentration of 2.6 × 10^9^ copies/μL.

### 3.2. sgRNA Screening

**Figure 2:**
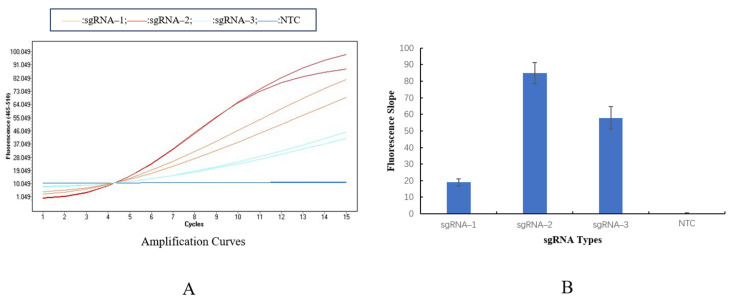
Fluorescence detection results of sgRNA screening. Note: (**A**): Fluorescence amplification curves of sgRNAs. (**B**): Comparison of fluorescence values of the three sgRNAs.

The fluorescence detection results of the three sgRNAs showed (Figure 2A) that the fluorescence intensity of sgRNA-2 (84.97 ± 2.31) was clearly higher than that of sgRNA-1 (18.97 ± 1.25) and sgRNA-3 (57.50 ± 1.89), and its signal increased at the fastest rate. The fluorescence intensity of the negative control (NTC) was only 0.376 ± 0.05, with no obvious signal amplification (Figure 2B). Therefore, sgRNA-2 was determined as the optimal guide RNA.

### 3.3. Optimization of the RAA System

**Figure 3:**
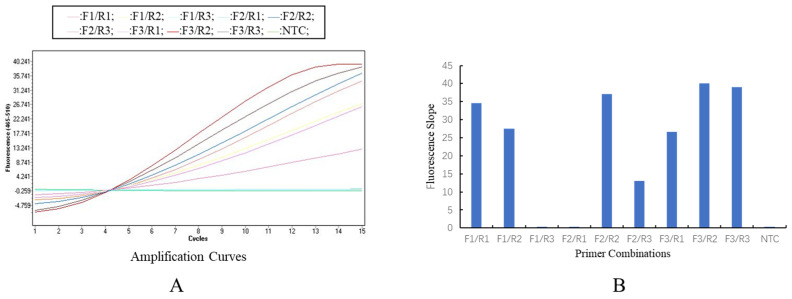
Screening of RAA primers. (**A**): Fluorescence amplification curves of different RAA primer pairs. (**B**): Fluorescence growth rates of different RAA primer pairs.

The fluorescence curves and fluorescence growth rates of the 9 primer combinations showed (Figure 3A,B) that the RAA-F3/R2 combination had the steepest slope (39.996 ± 1.52), which was higher than that of other combinations. Thus, it was determined as the optimal primer pair.

**Figure 4:**
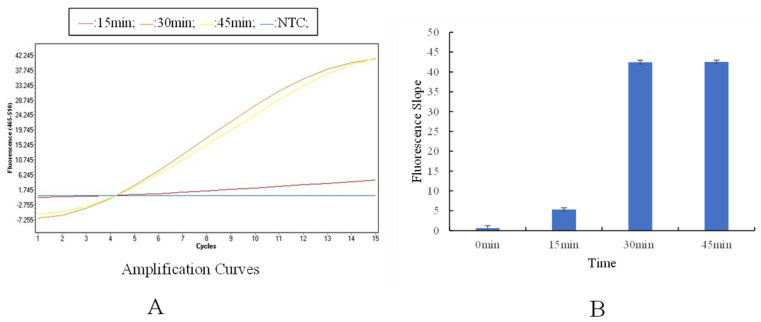
Screening of RAA reaction time. (**A**): Fluorescence amplification curves of the RAA system at different reaction times. (**B**): Fluorescence growth rates of the RAA system at different reaction times.

Optimization of reaction time: When the RAA reaction time was 30 min, the fluorescence intensity reached 42.045 ± 1.21, which showed no obvious difference from that at 45 min (42.137 ± 1.18), but was clearly higher than that at 15 min (Figure 4). Therefore, 30 min was determined as the optimal reaction time for RAA.

### 3.4. Validation Results

#### 3.4.1. Specificity

**Figure 5:**
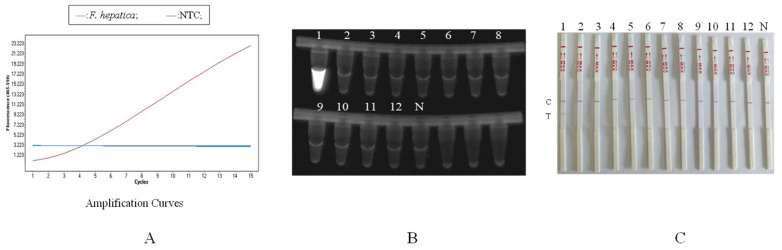
(**A**): Real-time fluorescence detection curves. Only the red line representing *F. hepatica* exhibited a specific positive fluorescent signal. All other tested parasite species (including *F. gigantica*, *F. jacksoni*, *P. cervi*, *D. dendriticum*, *E. pancreaticum*, *T. multiceps*, *T. crassiceps*, *E. granulosus*, *H. contortus*, *C. parvum*, *G. duodenalis*) and the no-template control (NTC, blue line) showed no amplification, with their curves overlapping with the baseline and thus indistinguishable in the figure. (**B**): UV lamp detection method. (**C**): Test strip detection (1: *F. hepatica*, 2: *F. gigantica*, 3: *F. jacksoni*, 4: *P. cervi*, 5: *D. dendriticum*, 6: *E. pancreaticum*, 7: *T. multiceps*, 8: *T. crassiceps*, 9: *E. granulosus*, 10: *H. contortus*, 11: *C. parvum*, 12: *G. duodenalis*, N: negative, C: control line; T: test line).

The RAA-CRISPR/Cas12b method was used to detect *F*. *hepatica*, *F*. *gigantica*, *F*. *jacksoni*, *P. cervi*, *D. dendriticum*, *E. pancreaticum*, *T. multiceps*, *T. crassiceps*, *E. granulosus*, *H. contortus*, *C. parvum and G. duodenalis*. The results showed that only the *F*. *hepatica* samples exhibited specific fluorescent signals for positive samples, while no specific amplification signals were detected in other genomic samples and the NTC (Figure 5A). UV lamp detection showed that only the *F*. *hepatica* samples had obvious fluorescence, while the other samples showed no fluorescence (Figure 5B). The test-strip detection results showed that only the *F*. *hepatica* samples showed both the C-line and the T-line, while the other samples only showed the C-line (Figure 5C), indicating that this method had good specificity and no cross-reactivity.

#### 3.4.2. Sensitivity

**Figure 6:**
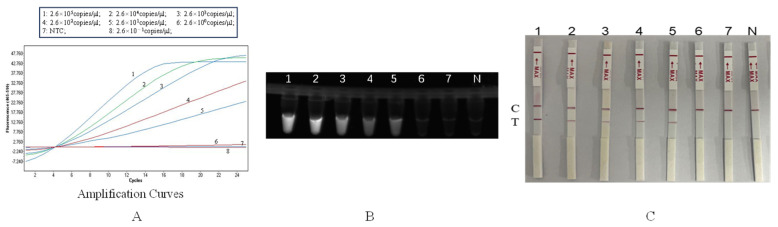
Sensitivity experiment. (**A**): Real-time fluorescence detection curve; (**B**): UV lamp detection method; (**C**): Test strip detection (1: 2.6 × 10^5^ copies/μL, 2: 2.6 × 10^4^ copies/μL, 3: 2.6 × 10^3^ copies/μL, 4: 2.6 × 10^2^ copies/μL, 5: 2.6 × 10^1^ copies/μL, 6: 2.6 × 10^0^ copies/μL, 7: 2.6 × 10^−1^ copies/μL, N: Negative, C: control line; T: test line).

The plasmid template was serially diluted by ten-fold gradients to concentrations of 2.6 × 10^5^ copies/μL–2.6 × 10^−1^ copies/μL. Detected by the RAA-CRISPR/Cas12b method, the limit of detection (LOD, copies/μL) of the real-time fluorescence detection method for the plasmid was 2.6 copies/μL (Figure 6A), that of the UV lamp method was 2.6 × 10 copies/μL (Figure 6B), and that of the test strip method was 2.6 × 10 copies/μL (Figure 6C).

#### 3.4.3. Clinical Sample Detection

**Figure 7:**
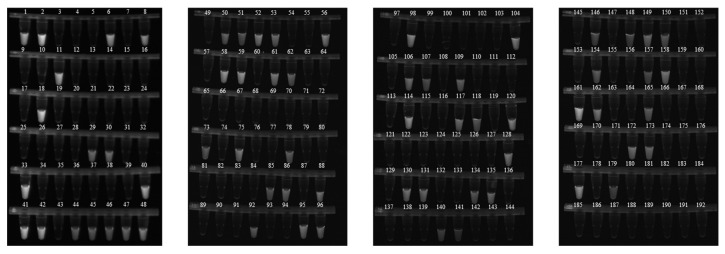
Diagnosis of Clinical Samples by RAA–CRISPR/Cas12b.

The test results of 192 clinical fecal samples indicated that the detection positive rate of the RAA-CRISPR/Cas12b method was 66 out of 192 (Figure 7). The results of PCR and qPCR were concordant, with 64 positive cases out of 192. Samples No. 73 and No. 141 were negative in PCR and qPCR but positive in RAA-CRISPR/Cas12b. Overall, the RAA-CRISPR/Cas12b method demonstrated a 98.95% (190/192) concordance with PCR and qPCR. Moreover, the total detection time of this method (30 min RAA + 15 min CRISPR) was merely 45 min, which was significantly shorter than that of conventional PCR (2.5 h). These results suggest that the RAA-CRISPR/Cas12b system is feasible and reliable for the detection of *F*. *hepatica* samples.

## 4. Discussion

Effective prevention and control of fascioliasis rely on early and accurate diagnostic techniques. In this study, a rapid detection method for *F*. *hepatica* was successfully established based on the RAA-CRISPR/Cas12b technologies. Moreover, an innovative test-strip visualization system was introduced [22], achieving the integration of “isothermal amplification-specific detection-on-site interpretation”, which provides a new solution for the clinical diagnosis of fascioliasis.

The core innovation of this study lies in the combination of the “rapid amplification” of RAA and the “precise recognition” of CRISPR/Cas12b. Meanwhile, the detection performance was further enhanced through the design of sgRNA and the optimization of the RAA system. In the design of sgRNA, the mitochondrial DNA (MT862417.1) of *F*. *hepatica* was selected as the target. This gene is characterized by a high copy number (thousands of copies per cell) and evolutionary conservation (homology > 95% among different geographical strains) [23], which can effectively improve the detection sensitivity. At the same time, through sequence alignment, it was ensured that the Spacer region of sgRNA-2 had a homology of <65% with that of the host and related parasites, and it strictly followed the A/T base preference at the 3′-end of Cas12b [24]. Ultimately, specific recognition of only *F*. *hepatica* was achieved with no cross-reactivity, solving the false-positive issue caused by antigen homology in traditional ELISA.

In terms of the optimization of the RAA reaction system, the screened primer pair RAA-F3/R2 can complete efficient isothermal amplification within 30 min. This result is highly consistent with the isothermal amplification characteristics of the RAA technology. Different from PCR/qPCR that rely on precise thermocycling equipment, the recombinase in the RAA system can rapidly mediate the binding of primers to the target and initiate amplification at a constant temperature [25]. This mechanism that does not require thermal cycling lays the foundation for the subsequent development of portable detection devices. Regarding the detection sensitivity, the minimum detection limit of the fluorescence detection method reaches 2.6 copies/μL, while that of the UV lamp method and the test-strip method is 2.6 × 10 copies/μL. This sensitivity level is superior to that of traditional PCR detection (0.5 ng/μL) [21] and qPCR detection (1 pg/μL) [20], and is also comparable to the sensitivity of the CRISPR-mediated parasite detection systems reported in previous studies [26]. This benefits from the dual signal-amplification effects of the efficient isothermal amplification of RAA and the trans-cleavage of Cas12b. It also indicates that this method can effectively detect low-load samples in the early stage of infection, compensating for the insufficient sensitivity of traditional fecal egg microscopy. In terms of specificity evaluation, the RAA-CRISPR/Cas12b system has been verified for cross-reactions with various trematodes and other common parasites. It specifically recognizes only *F*. *hepatica*, with no non-specific amplification. Its verification scope is better than that of traditional PCR and qPCR, providing a reliable guarantee for the detection of clinical complex samples.

Notably, two clinical samples tested positive by the RAA-CRISPR/Cas12b assay but negative by PCR/qPCR. Considering the strict design of sgRNA targeting the PAM site of Cas12b and the absence of cross-reactivity in the specificity test, false-positive results due to non-specific binding are highly unlikely. Instead, these discrepancies may be attributed to the higher sensitivity of the current system in detecting low-template DNA in clinical specimens from naturally infected grazing sheep in endemic areas. After DNA extraction, they were stored in a −20 °C frozen environment, with no nucleic acid degradation or cross-contamination. The sample background has a real possibility of infection. In terms of analytical sensitivity, the RAA-CRISPR/Cas12b fluorescence assay was more sensitive than traditional PCR/qPCR. It is speculated that these 2 samples are likely to be low-parasite-load samples in the early stage of infection. Although their nucleic acid concentrations are lower than the detection thresholds of PCR/qPCR, they can be effectively captured by the efficient isothermal amplification of RAA and the trans-cleavage signal-amplification system of Cas12b. This result precisely confirms the advantage of this method in the detection of low-load infections rather than being caused by false positives. The detection sensitivity of the test-strip method is one order of magnitude lower than that of the fluorescence method. This gap mainly stems from the differences in the signal transduction mechanisms of the two read-out modes. The fluorescence method relies on the real-time cumulative fluorescence signal, which can capture the trace amounts of trans-cleavage products triggered by low-concentration targets. In contrast, the test-strip method requires a sufficient amount of cleaved ssDNA reporter molecules to accumulate during chromatography to form a visible band. When the target concentration is low, the number of labeled nucleic acid molecules generated by trans-cleavage is limited, making it difficult to form a strong enough signal precipitate on the test line. This limitation has also been reported in other CRISPR-based test-strip detection studies [27].

In addition, the detection performance of the test strip is easily affected by the fecal sample matrix. Fecal samples have complex matrix components. Impurities such as residual proteins, polysaccharides, and humic acid in them may non-specifically adsorb onto the surface of the nitrocellulose membrane or cross-bind with the immobilized antibodies/nucleic acid probes, thus interfering with the signal formation on the test line and further weakening the detection ability for low-abundance targets [28]. This matrix effect is relatively common in rapid detection technologies based on the lateral flow principle and is one of the main bottlenecks for highly sensitive detection directly from complex biological samples.

Meanwhile, the inter-batch differences in the test-strip production process cannot be ignored. Factors such as probe immobilization density, label release efficiency, and membrane pore-size uniformity may all affect the analytical performance and stability of the test strip [29]. The above-mentioned problems will be further amplified in the resource-limited grassroots on-site detection scenarios, restricting its application in the diagnosis of early low-load infections of *F. hepatica*.

In the veterinary clinical setting, the “on-site applicability” of detection techniques often determines their practical application value. The stringent requirements for instrument dependence, technical complexity of operation, and detection rapidity in scenarios such as grass-roots veterinary stations and slaughterhouses make it difficult for traditional PCR (which depends on a thermal cycler and takes 2.5 h) and ELISA (which has a cumbersome operation and requires 3–4 h) to meet the needs. The RAA-CRISPR/Cas12b system established in this study breaks through this limitation through three technical features: First, the instrument dependence is minimized. RAA only requires a constant-temperature metal bath, and the visual interpretation of the test strip can be completely independent of instruments, solving the bottleneck in the popularization of molecular diagnostic techniques in resource-limited scenarios. Second, the total detection cycle of 45 min enables a same-day closed-loop from “sample collection-result reporting”, increasing the efficiency by 3–5 times compared to traditional methods. Third, after simplifying the operation process, grassroots personnel with minimal technical training can master it, significantly reducing the dependence on professional skills. Clinical verification shows that the concordance of the detection positive rate between this method and PCR is highly significant, confirming that it can be used as an alternative to conventional PCR. Although the sensitivity of the test-strip method is slightly lower than that of the fluorescence method (differing by one order of magnitude), it already covers the common parasite loads in clinical samples (>10^1^ copies/μL). In slaughterhouse surveillance, this method allows for the rapid identification of animals infected with *F. hepatica*. Although infected livers do not directly transmit fascioliasis, early detection during slaughter can help assess the prevalence of infection in the herd, provide important data for regional prevention and control, reduce economic losses caused by liver condemnation, and lower the risk of environmental contamination by preventing infected animals from shedding eggs into pastures and water sources. Nevertheless, our study has two main limitations. First, the test strip and UV lamp methods exhibit one order of magnitude lower sensitivity than the fluorescence assay, with a detection limit of 2.6 × 10 copies/μL, which may cause false-negative results in low-pathogen-load samples during early infection. Second, fecal sample processing still depends on commercially available genomic DNA extraction kits, and completely instrument-free on-site detection has not been fully realized. These issues will be the focus of subsequent technical optimization.

Compared with the previously reported CRISPR-based parasite detection systems (such as the *Plasmodium* Cas12a method and the *Schistosoma* Cas13a system) [30], the innovative value of this study lies in the in-depth integration of technical specificity and scenario adaptability. This is the first application of the Cas12b system to the detection of *F. hepatica*. Its strict TTN PAM recognition feature (different from the TTN/TTTT compatible mode of Cas12a) significantly reduces the off-target risk [31], providing a higher guarantee of specificity for the detection of complex samples (such as mixed-infection tissues containing multiple parasites). The combination of test-strip visualization and systematic verification of clinical applicability not only fills the technical gap in the on-site rapid diagnosis of *F. hepatica*, but also provides a reusable implementation paradigm for the transformation of CRISPR technology from the laboratory to veterinary clinical practice [32].

Currently, this method has three aspects that warrant further improvement. The sensitivity of the test strip is limited by the probe immobilization efficiency and signal amplification ability. This can be optimized by replacing traditional fluorescent dyes with quantum dot labeling and selecting a high-affinity nitrocellulose membrane to improve the chromatography efficiency. The grinding-extraction steps in sample pretreatment restrict the detection speed, and a “no-extraction” reaction system needs to be developed (such as adding proteinase K and nucleic acid release agents to directly lyse the parasites [16]). Single-target detection is difficult to deal with common mixed infections in cattle and sheep (such as *F. hepatica* + *H. contortus*) [26]. It is necessary to construct multi-sgRNA combinations and multi-channel test strips (with different test lines labeled with specific fluorescence) to achieve “multi-detection in one tube” for multiplex diagnosis.

In terms of practical applications, the established detection method can be used not only for the clinical diagnosis of fascioliasis in cattle and sheep, but also extended to multiple directions such as epidemiological surveillance, auxiliary diagnosis of human fascioliasis, and evaluation of drug efficacy. For example, in endemic areas, large-scale screening can be rapidly carried out through test strips, providing a technical means to understand the infection distribution and epidemic trends. In regions with a high incidence of human fascioliasis, this method can also serve as an effective supplement to traditional etiological detection, improving the primary screening efficiency. In addition, regularly monitoring the changes in parasite load after drug treatment helps to objectively evaluate the efficacy and guide clinical medication. It is worth noting that this technical framework has good versatility and can be further extended to the detection of other important veterinary parasites such as *Toxoplasma gondii* and *Cryptosporidium* spp. in the future, demonstrating considerable practical application potential.

## 5. Conclusions

In this study, a rapid detection method for *F*. *hepatica* based on RAA-CRISPR/Cas12b is established. This method integrates three detection modes: real-time fluorescence, UV lamp, and test strip. It features high specificity, high sensitivity, rapidity, portability, and strong clinical applicability. This method provides a reliable tool for the laboratory-supported on-site diagnosis and epidemiological surveillance of fascioliasis. With the use of a commercial DNA extraction kit, the established assay demonstrates considerable application potential in veterinary clinical practice and also provides a useful reference for the development of diagnostic techniques for other parasitic diseases.

## Figures and Tables

**Table 1:** Oligo sequences used in the studies.

Name	Sequence (5′-3′)
Fh603F	TCTTTTTCGGTTCCGGAGTT
Fh603R	ACAGTAAGACAAACCCTCAAACCT
FH-sgRNA-1	GUCUAAAGGACAGAUUUUCAACGGGUGUGCCAAUGGCCACUUUCCAGGUGGCAAAGCCCGUUGAACUUCAAGCGAAGUGGCAC**GUUGUAGGGUUCAGUUGAUA**
FH-sgRNA-2	GUCUAAAGGACAGAUUUUCAACGGGUGUGCCAAUGGCCACUUUCCAGGUGGCAAAGCCCG UUGAACUUCAAGCGAAGUGGCAC**GUUUAUG UGGGUGGCGUUUA**
FH-sgRNA-3	GUCUAAAGGACAGAUUUUCAACGGGUGUGCCAAUGGCCACUUUCCAGGUGGCAAAGCCCGUUGAACUUCAAGCGAAGUGGCAC**AGUUGAUAUUUAGUGGUUUU**
FH-PCR-F	CCCATAGTTTATTGTTTGTTG
FH-PCR-R	CCTCTAAATTTAGGTAATGGATTG
RAA-F1	GGTTGTAGGGTTCAGTTGATATTTAGTGGT
RAA-F2	TATTTGGTTGTAGGGTTCAGTTGATATTTA
RAA-F3	GGTGCTGCTTTAAGTATTTCTGGTTTGGGT
RAA-R1	ATTGGGGGTATGAATGGAAACAAAAATAAA
RAA-R2	AGGTAATGGATTGGGGGTATGAATGGAAAC
RAA-R3	GAACCAGCCCATCAATCCCAATACCTCTAA

Note: The boldfaced letters represent the sgRNA spacer sequence.

## Data Availability

The original contributions presented in the study are included in the article, further inquiries can be directed to the corresponding authors.
